# Potential Iatrogenic Harms From Medicinal Cannabis Prescribing in Australia: A Case Series

**DOI:** 10.1111/dar.70255

**Published:** 2026-08-31

**Authors:** Richard Bradlow, James Sorensen, Anna Cunningham, Sean Daugherty, Eric Hadinata, Ferghal Armstrong, Nazila Jamshidi

**Affiliations:** ^1^ Department of Psychiatry Monash University Melbourne Australia; ^2^ The Victoria Clinic Melbourne Australia; ^3^ Department of Psychiatry Alfred Hospital Melbourne Australia; ^4^ Department of Psychiatry St Vincent's Hospital Melbourne Australia; ^5^ Department of Addiction Eastern Health Melbourne Australia; ^6^ Department of Addiction Royal Melbourne Hospital Melbourne Australia

## Abstract

**Introduction:**

Medicinal cannabis prescribing in Australia has increased markedly, including for psychiatric indications where evidence of benefit is limited and of low certainty. The rise of highly commercialised prescribing models has raised concerns regarding patient safety, particularly for those with pre‐existing psychiatric or addiction vulnerabilities.

**Case Presentation:**

This case series describes three young men (aged 20–24) with psychiatric disorders and substance use vulnerabilities who were prescribed medicinal cannabis. The cases illustrate potential iatrogenic harms, including cannabinoid hyperemesis syndrome, cannabis use disorder, paranoia, affective instability and the delaying of other treatment. In two cases, prescribing continued despite emerging adverse outcomes or a formal request for cessation of prescribing from an addiction specialist.

**Discussion and Conclusion:**

These cases highlight the potential iatrogenic harms associated with the current medicinal cannabis prescribing practices in Australia, especially among those with psychiatric and substance use vulnerabilities. More rigorous, evidence‐aligned prescribing, clearer exclusion criteria and a lower threshold for discontinuation when harms emerge are needed.

## Key Point Summary

- Evidence Gap: Despite limited evidence for its efficacy in treating psychiatric disorders, medicinal cannabis is frequently prescribed in Australia for anxiety, depression and attention‐deficit/hyperactivity disorder.
- Vulnerability Risks: In people with pre‐existing psychiatric conditions or a history of substance use disorders, medicinal cannabis may contribute to the development or continuation of cannabis use disorder or other psychiatric and physical harms, including cannabinoid hyperemesis syndrome.
- Commercial prescribing: These cases raise concern that the current model of commercial prescribing may lead to the prioritisation of access over comprehensive assessment, monitoring and an appropriate response to emerging clinical harms.
- Clinical Recommendation: Stricter screening for current and previous substance use and psychiatric disorders, clearly defined evidence‐aligned indications, close monitoring for adverse effects and cannabis use disorder and explicit thresholds for discontinuation are recommended.

## 1 Introduction

Since regulatory access to medicinal cannabis expanded in Australia in 2016, prescribing has increased markedly [1]. This expansion has occurred alongside the growth of a highly commercialised prescribing sector, including dedicated medicinal cannabis clinics [2]. Anxiety disorders and insomnia remain among the most common indications, despite limited and low‐certainty of benefit [3]. Ongoing use of the special access scheme without Therapeutic Goods Administration‐approved indications has enabled high‐volume prescribing for conditions with weak evidence, potentially delaying access to evidence‐based treatments and in some cases contributing to cannabis‐related harms.

While some patients may experience symptomatic benefit, evidence for efficacy in most psychiatric disorders remains limited and heterogeneous [3]. This case series describes three patients in whom medicinal cannabis was associated with harm, with clinical improvement following cannabis cessation alongside addiction treatment. All three presented to the same metropolitan addiction clinic in 2024 and 2025 for treatment of cannabis use disorder (CUD) while being prescribed medicinal cannabis. Formal ethics approval was not required for case reports. All patients provided written informed consent for publication of their anonymised clinical information. CUD was diagnosed clinically by treating addiction specialists with reference to DSM‐5 criteria. No structured diagnostic instruments were administered because the diagnoses arose during routine clinical care. Outcomes were based on patient self‐report and treating clinician assessment rather than validated rating scales.

### 1.1 Case 1

AB was a 24‐year‐old man with a history of attention‐deficit/hyperactivity disorder (ADHD), depression and alcohol use disorder in remission, who presented to an addiction clinic with active CUD. Prior to the prescription AB had not used cannabis. AB's CUD was characterised by tolerance, withdrawal, use despite it negatively affecting his physical health (cannabinoid hyperemesis syndrome [CHS]) and psychiatric health (he noted it exacerbated his anxiety), being unable to reduce/cease his use and intense cravings. AB saw a cannabis prescriber in person. The stated initial indication was for the management of anxiety and insomnia. Despite his known history of alcohol use disorder, he was prescribed medicinal cannabis via a vaporiser (various tetrahydrocannabinol [THC]/cannabidiol [CBD] strengths), which was associated with the development of CUD. AB then developed CHS, for which he sought help from his cannabis prescriber and was inappropriately advised to continue his cannabis prescription and simply change the cannabis strength.

AB consulted an addiction specialist who arranged an inpatient admission for withdrawal treatment. AB had been prescribed cannabis for 15 months prior to seeing the addiction specialist. The diagnosis of CHS was made by his cannabis prescriber and re‐confirmed by the addiction specialist. His CHS presented with severe nausea and vomiting relieved by hot showers, which resolved with the cessation of cannabis.

Following withdrawal treatment and cannabis cessation, AB reported an improved social life and physical health and reduced anxiety. He has been predominantly abstinent for more than 2 years, with two brief episodes of cannabis use.

### 1.2 Case 2

CD was a 24‐year‐old male with a history of bipolar affective disorder, generalised anxiety disorder and ADHD. Bipolar affective disorder was recorded at the time of prescribing. He was prescribed 10 g of medicinal cannabis flower (THC/CBD ratios 26% and 22%/< 1%) per fortnight. This prescription was associated with the subsequent development of CUD. Prior to the prescription, CD had used cannabis but had not engaged in regular use. CD's CUD was characterised by tolerance, withdrawal, use despite it negatively affecting his psychiatric health (he noted it exacerbated his anxiety), being unable to reduce/cease his use and intense cravings. CD saw a cannabis prescriber via telehealth only. The stated indication was for the management of anxiety.

CD sought help from an addiction psychiatrist who facilitated an inpatient admission for withdrawal treatment. CD had been prescribed cannabis for 7 months prior to seeing the addiction psychiatrist. His diagnosis was later reformulated from bipolar affective disorder to borderline personality disorder. With psychotherapy, targeted pharmacotherapy and sustained cannabis abstinence, his anxiety and overall functioning improved; he returned to work, exercised regularly and self‐reported a marked reduction in his anxiety. After the addiction specialist had requested cessation, CD received a reorder reminder 2 months into abstinence, which he declined. CD has remained abstinent from cannabis for more than 6 months.

### 1.3 Case 3

EF was a 20‐year‐old male with a history of complex post‐traumatic stress disorder, depression and generalised anxiety disorder. He had a pre‐existing CUD from the age of 15 before transitioning to prescribed cannabis flower (THC/CBD ratio 20%/< 1%), prescribed 21 g a fortnight. Over time, EF developed severe paranoia, panic attacks and likely CHS in the context of ongoing high THC cannabis exposure. Although he recognised his addiction, he felt unable to discuss cessation with his prescribing doctor due to anxiety. EF engaged an addiction psychiatrist to advocate on his behalf and write to his prescriber. The diagnosis of CHS was made clinically by the psychiatrist. His CHS presented with severe nausea and vomiting relieved by hot showers, which resolved with the cessation of cannabis.

EF had been prescribed cannabis for 21 months prior to seeing the addiction psychiatrist. When EF engaged with the addiction psychiatrist, his CUD was characterised by tolerance, withdrawal, use despite it negatively affecting his physical health (CHS) and psychiatric health (psychosis), being unable to reduce/cease his use and intense cravings. EF saw a cannabis prescriber via telehealth only. The stated indication was for the management of anxiety. Following psychotherapy, targeted pharmacotherapy and abstinence from cannabis, EF's mental state and quality of life substantially improved; he exercised regularly, described improved mood and everyday functioning and reported resolution of his paranoia and panic attacks. EF is now over 1 year abstinent from cannabis.

## 2 Discussion

This case series describes three cases in which patients improved with treatment and the cessation of medicinal cannabis. All three patients had pre‐existing vulnerabilities (psychiatric illnesses and substance use disorders). In all cases, prescribing was continued despite concern for possible serious side effects of cannabis (CHS, CUD, psychotic symptoms) that would ordinarily prompt caution, review or discontinuation. Case 1's concerns about CHS were not appropriately addressed, potentially prolonging harm; Case 2 received a reorder reminder 2 months into abstinence after the addiction specialist had requested cessation; and Case 3 felt unable to ask for cessation. In Cases 1 and 2, CUD developed after commencement of prescribed cannabis, supporting a temporal relationship and the possibility of an iatrogenic contribution. However, the case‐series design cannot establish causality. In Case 3, prescribing did not initiate CUD but provided continued access to high‐THC cannabis in the context of an established disorder. Across the three cases, improvement followed cannabis cessation alongside other interventions, including withdrawal treatment, psychotherapy, pharmacotherapy, inpatient care and diagnostic reassessment; the independent contribution of cannabis cessation cannot be isolated.

The current trend in prescribing is increasingly difficult to reconcile with the existing evidence. A recent large review found limited and low‐certainty evidence supporting the use of cannabinoids in the management of most psychiatric or substance use disorders [4]. Any possible benefits were restricted to a small number of indications and supported by low‐quality evidence. These findings are particularly alarming in Australia, where psychiatric conditions account for a substantial proportion of medicinal cannabis approvals and prescribing for anxiety, sleep disorders, ADHD and depression has expanded [5]. CUD is common among people who use medicinal cannabis. An international meta‐analysis estimated a pooled prevalence of approximately 25% and an Australian survey found that 41% of people using prescribed medicinal cannabis met DSM‐5 criteria for CUD, with 15% meeting criteria for moderate‐to‐severe CUD. Risk was greater in those who use medicinal cannabis daily, use larger quantities, use via inhalation, are younger, are male and have another substance use disorder [1]. The Dawson meta‐analysis pooled 14 studies involving 3681 participants across five countries. Among people using cannabis for medicinal reasons, the pooled prevalence of DSM‐5 CUD was 25% (95% confidence interval 18% to 33%); this was not an Australia‐specific estimate. More frequent cannabis use, younger age and male sex were associated with higher risk. Calls to the NSW Poisons Information Centre concerning cannabis exposures increased during 2014–2024; however, the exposures cannot be attributed specifically to prescribed products [2].

Both the Australian academic literature [6, 7] and news media [3] have raised considerable concerns about the clinical consequences of this prescribing environment. These cases raise concerns about the adequacy of assessment, monitoring and support for discontinuation in some commercial and telehealth prescribing settings; telehealth alone does not establish inadequate care [6]. While some patients may derive benefit from medicinal cannabis, particularly in non‐psychiatric indications, these cases highlight potential risks in vulnerable populations. These cases raise concerns about the adequacy of assessment, monitoring and support for discontinuation in these prescribing settings.

Irrespective of broader regulatory reform, prescribers can take immediate steps to reduce harm. The Special Access Scheme and Authorised Prescriber pathways provide access to unapproved therapeutic goods. Before initiating medicinal cannabis, clinicians should document an evidence‐aligned indication and treatment goals, assess current and previous substance use disorders and psychiatric disorders, review relevant clinical records and check real‐time prescription monitoring systems for Schedule 8 products where available and applicable. Patients should receive psychoeducation regarding CUD, withdrawal, CHS, psychiatric deterioration, cognitive impairment and driving risk, with early and regular follow‐up. Emerging CUD or psychiatric or physical harm should prompt reassessment of the indication, supported dose reduction or cessation, coordination with the patient's other treating clinicians and referral to addiction services when required. Suspected adverse events should be reported to the Therapeutic Goods Administration.

This case series has several limitations. The three patients were identified within a single specialist addiction service and are therefore subject to referral and selection bias; the findings cannot estimate the prevalence of adverse outcomes among people prescribed medicinal cannabis. The clinical histories were retrospectively obtained. The precise timing of symptom onset and the interval between cannabis cessation and subsequent improvement could not always be determined reliably from the available records. Diagnoses and outcomes were assessed clinically rather than using structured diagnostic interviews or validated outcome measures. Improvement followed cannabis cessation alongside other interventions, including withdrawal treatment, psychotherapy, pharmacotherapy, inpatient care and diagnostic reassessment; the independent contribution of cannabis cessation cannot be isolated. Finally, the cases cannot establish whether the harms resulted from cannabis exposure, high‐THC products, individual prescribing decisions or broader features of the Australian prescribing system.

These cases add to the existing literature about medicinal cannabis prescribing in Australia. In all three cases, prescribing was temporally associated with physical and psychiatric harms and may have contributed to them. Importantly, all three cases improved after engaging in evidence‐based addiction and psychiatric treatment alongside cannabis cessation. Prescribing may have delayed access to evidence‐based treatment, although this cannot be established from this case series.

The system would benefit from more rigorous screening for CUD and serious mental illness, clearer exclusion criteria, closer follow‐up, a lower threshold for discontinuation, and, most importantly, strictly defined clinical indications for prescriptions. Further investigations are required to better inform safe prescribing practices and to characterise the risks inherent within the current prescribing system. These cases highlight the ongoing need for a more cautious and evidence‐aligned approach to cannabis prescribing in psychiatric populations.

## Author Contributions

R.B. conceptualised the article and drafted the initial manuscript. All authors (R.B., J.S., A.C., S.D., E.H., F.A. and N.J.) critically revised the manuscript for intellectual content, gave final approval of the version to be published and agree to be accountable for all aspects of the work.

## Funding

The authors have nothing to report.

## Conflicts of Interest

The authors declare no conflicts of interest.

## Data Availability

Data sharing not applicable to this article as no datasets were generated or analysed during the current study.
